# White Matter Microstructure Alterations Associated With Paroxetine Treatment Response in Major Depression

**DOI:** 10.3389/fnbeh.2021.693109

**Published:** 2021-07-22

**Authors:** Rita Vieira, Ana Coelho, Joana Reis, Carlos Portugal-Nunes, Ricardo Magalhães, Sónia Ferreira, Pedro Silva Moreira, Nuno Sousa, João M. Bessa

**Affiliations:** ^1^Life and Health Sciences Research Institute (ICVS), School of Medicine, University of Minho, Braga, Portugal; ^2^ICVS/3B’s, PT Government Associate Laboratory, Braga/Guimarães, Portugal; ^3^Clinical Academic Center – Braga, Braga, Portugal; ^4^NeuroSpin, Institut des Sciences du Vivant Frédéric Joliot, Commissariat à l’ Énergie Atomique et aux Énergies Alternatives, Université Paris-Saclay, Gif-sur-Yvette, France; ^5^Psychological Neuroscience Laboratory, CIPsi, School of Psychology, University of Minho, Braga, Portugal

**Keywords:** depression, paroxetine, SSRI, treatment response, diffusion MRI, white-matter, tract-based spatial statistics

## Abstract

More than one-third of depressive patients do not achieve remission after the first antidepressant treatment. The “watch and wait” approach used to find the most effective antidepressant leads to an increased personal, social, and economic burden in society. In order to overcome this challenge, there has been a focus on studying neural biomarkers associated with antidepressant response. Diffusion tensor imaging measures have shown a promising role as predictors of antidepressant response by pointing to pretreatment differences in the white matter microstructural integrity between future responders and non-responders to different pharmacotherapies. Therefore, the aim of the present study was to explore whether response to paroxetine treatment was associated with differences in the white matter microstructure at baseline. Twenty drug-naive patients diagnosed with major depressive disorder followed a 6- to 12-week treatment with paroxetine. All patients completed magnetic resonance brain imaging and a clinical assessment at baseline and 6–12 weeks after treatment. Whole-brain tract-based spatial statistics was used to explore differences in white matter microstructural properties estimated from diffusion magnetic resonance imaging. Voxel-wise statistical analysis revealed a significant increase in fractional anisotropy and a decrease in radial diffusivity in forceps minor and superior longitudinal fasciculus in responders compared to non-responders. Thus, alterations in white matter integrity, specifically in forceps minor and the superior longitudinal fasciculus, are associated with paroxetine treatment response. These findings pave the way for personalized treatment strategies in major depression.

## Introduction

Major depressive disorder (MDD) is one of the major contributors to the overall global burden of disease, affecting nearly 300 million people in 2019 (Vos et al., 2020). According to the American Psychiatric Association (APA) clinical guidelines, there are several approved treatments for MDD, specifically psychotherapy, pharmacotherapy, combination of both psychotherapy and pharmacotherapy, and other interventions, such as electroconvulsive therapy (ECT) and transcranial magnetic stimulation (TMS) (Gelenberg et al., 2010).

Pharmacotherapy is recommended as an initial treatment for patients with mild to severe MDD symptoms (Gelenberg et al., 2010). Selective serotonin reuptake inhibitors (SSRIs) are usually the first-line treatment choice in clinical practice; however, and despite their clinical relevance, only 60% of MDD patients respond to the first treatment (Gartlehner et al., 2011), and from those, only 36.8% remit (Rush et al., 2006). The remaining patients start a long process of successive trials until finding the most effective treatment, but the remission percentage decreases as the number of treatment trials increase (e.g., 13.7% for the third treatment) (Rush et al., 2006).

The current challenge for clinicians is not the lack of effective treatments, but the choice of the most effective antidepressant for each patient. As there are no objective measures to guide treatment choice, clinicians use the standard approach of “watch and wait” based on close observation of patients for 4–12 weeks (Gelenberg et al., 2010). The period of wait repeats every time there is a new medication trial, extending the length of depressive episodes, consequently enhancing the burden of the disease, and increasing healthcare costs (Leuchter et al., 2009).

Prompted by this context, there has been a focus on identifying neurobiological predictors of pharmacological response by using different magnetic resonance imaging (MRI) modalities, such as structural, functional, and diffusion MRI. These techniques also enable the characterization of brain differences between responders and non-responders, which may lead to better patient prognosis and care.

Alterations in the white matter (WM) microstructure have been linked to antidepressant treatment response and remission in studies using diffusion tensor imaging (DTI). DTI indirectly assesses the WM microstructure properties using simple quantitative measures of the rate and directionality of the water molecule diffusion (Van Hecke et al., 2016). The measures, commonly derived from the DTI tensor, are fractional anisotropy (FA), axial diffusivity (AD), mean diffusivity (MD), and radial diffusivity (RD). FA, the most popular measure, provides information about the degree of anisotropic diffusion. Increased FA values indicate higher WM integrity (Alexander et al., 2011). MD measures the average diffusion rate, and lower values are associated with higher WM integrity (Alexander et al., 2011). AD and RD are defined as the parallel and perpendicular diffusivity to the main direction of the tract, respectively. The former might indicate axonal integrity (Song et al., 2002, 2003; Budde et al., 2007), whereas the latter is associated with the degree of myelination (Song et al., 2002, 2003, 2005; Klawiter et al., 2011).

Davis et al. (2019) characterized the organization and integrity of the WM associated with antidepressant response by describing differences in the FA, MD, AD, and RD between responders and non-responders to escitalopram treatment. It was reported that responders to escitalopram treatment had increased AD in the left external capsule, part of the superior longitudinal fasciculus (SLF), compared to non-responders and controls. Further analysis revealed decreased FA in the corona radiata and sagittal stratum and increased MD and RD in the cingulate portion of the cingulum bundle in non-responders. This comprehensive study suggested a disruption in WM integrity for non-responders to SSRI treatment (Davis et al., 2019). Other studies reported decreased FA of the left hippocampal part of the cingulum bundle in non-responders to citalopram or quetiapine treatment (Tatham et al., 2017), as well as in the WM tracts connecting the raphe nuclei to the amygdala in non-remitters to escitalopram treatment (DeLorenzo et al., 2013), and increased FA in the superior frontal and anterior cingulate cortices associated with non-remission to sertraline (Taylor et al., 2008).

Other studies explored the role of fronto-limbic WM tracts as potential predictors of treatment response and remission in MDD (Korgaonkar et al., 2014; Grieve et al., 2016). Non-remission to antidepressant treatment (escitalopram, sertraline, or venlafaxine-XR) was predicted by a high ratio of FA in the cingulate portion of the cingulum bundle and the stria terminalis. Despite its high specificity (83–88%), it only identified 29% of non-remitters to one of three antidepressant medications (Grieve et al., 2016).

Such findings are promising and represent progress in the identification of imaging biomarkers of treatment response in depression. However, we are still far from having a useful clinical measure to accurately predict response and remission to antidepressant treatments. The heterogeneous findings, which might be a consequence of using different analytical methods, regions of interest, or even antidepressant treatments, call for more studies in order to achieve useful clinical predictors of antidepressant response.

The present study aimed to explore whether response to paroxetine treatment was associated with differences in the WM microstructure at baseline. A sample of drug-naive patients diagnosed with MDD followed a 6- to 12-week treatment with paroxetine, completing an MRI acquisition and clinical assessments pre- and post-treatment.

## Materials and Methods

### Participants

Subjects were recruited at the emergency psychiatry department or the psychiatry outpatient unit of Hospital de Braga. To be eligible for this study, subjects had to be aged between 18 and 65 years, meet the Diagnostic and Statistical Manual of Mental Disorders, 4th edn., Text Revision (DSM-IV-TR) criteria for MDD without psychotic features assessed by an experienced psychiatrist through Structured Clinical Interview for DSM-IV (SCID) (First and Gibbon, 2004), and no prior history of antidepressant treatment (drug-naive). The exclusion criteria were any MRI contradictions, comorbid psychiatric disorders (e.g., bipolar disorder, addictive disorders, and schizophrenia), prior medical history of neurological disorders or traumatic brain injury, and any sign of cognitive impairment defined as Mini-Mental Sate Examination (MMSE) below 24 (Folstein et al., 1975).

**FIGURE 1:**
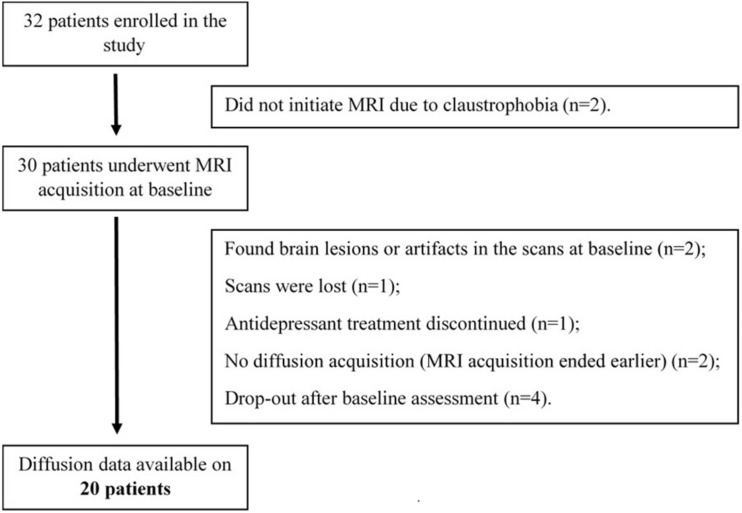
Detailed information on subject recruitment and exclusion. Thirty-two patients were enrolled in the study. From those, 30 patients completed MRI acquisitions and psychological assessment, but only 20 were included in the final analyses.

Following the aforementioned criteria, 32 patients were enrolled in the study between January 2016 and January 2020. From these, only 20 patients were included in the analysis (Figure 1). Information on the demographic and clinical data of these patients is displayed in Table 1.

**TABLE 1:** Demographic and clinical characterization of all patients (*N* = 20) included in the data analyses.

	***M* ± SD**
Age (years)	37.75 ± 12.29
Sex (male/female), *n*	6/14
Education (years)	11.40 ± 5.37
Time between assessments (weeks)	8.35 ± 1.69
Pretreatment HDRS score	21.20 ± 8.56
Pretreatment HARS score	23.20 ± 10.25
Pretreatment PSS-10 score	27.45 ± 5.34
Pretreatment BSSI score^a^	5.00 ± 6.99

*Data are presented as the mean ± standard deviation.*M*, mean; *SD*, standard deviation; *HDRS*, Hamilton Depression Rating Scale; *HARS*, Hamilton Anxiety Rating Scale; *PSS-10*, 10-item Perceived Stress Scale; *BSSI*, Beck Suicidal Ideation Scale. ^*a*^One participant with missing data.*

### Study Design and Clinical Measures

To reduce any confounds associated with multiple drug targets, we decided to focus on a single SSRI. Paroxetine was chosen because it is one of the most potent and selective SSRIs available (Thomas et al., 1987; Tulloch and Johnson, 1992; Nemeroff, 1994), with proven efficacy and effectiveness to treat MDD (Kroenke et al., 2001; Undurraga and Baldessarini, 2012). Moreover, no study, to our knowledge, has explored whether the response to paroxetine treatment was associated with differences in the WM microstructure at baseline.

All patients were drug-naive and initiated treatment with paroxetine (20 mg/day) after baseline evaluation. Brain MRI and clinical assessments were completed at baseline and 6–12 weeks after the beginning of the treatment. Clinical assessments included the Hamilton Depression Rating Scale (HDRS) (Hamilton, 1960) and the Hamilton Anxiety Rating Scale (HARS) (Hamilton, 1959) to evaluate both depressive and anxiogenic symptomatology, the Beck Scale for Suicidal Ideation (BSSI) (Beck and Steer, 1991) to evaluate suicidal ideation, and the 10-item Perceived Stress Scale (PSS-10) (Cohen and Williamson, 1988) in order to evaluate perceived stress. Response to treatment was defined as ≥50% reduction in the HDRS score from baseline to 6–12 weeks after treatment (Cusin et al., 2009).

### Diffusion MRI Acquisition

All patients underwent the same acquisition protocol using a clinically approved Siemens MAGNETOM Avanto 1.5T scanner (Siemens Medical Solutions, Erlangen, Germany) equipped with a 12-channel receive-only head coil. The imaging protocol included several different acquisitions, but only the diffusion-weighted imaging (DWI) acquisition was considered for the present study. DWI scans were performed using a spin echo–echo planar imaging (SE-EPI) sequence: TR = 8,800 ms, TE = 99 ms, FoV = 240 mm × 240 mm, acquisition matrix = 120 × 120, 61 two-millimeter axial slices with no gap, 30 non-collinear gradient directions with *b* = 1,000 s mm^–2^, one *b* = 0 s mm^–2^ acquisition, and one repetition.

Before data pre-processing, the raw acquisitions from all participants were visually inspected to discard any brain lesions, critical head motion, or artifacts that could compromise the data.

### DWI Image Pre-processing and Tensor Fitting

Diffusion data were pre-processed using the FMRIB Diffusion Toolbox (FDT) provided with the FMRIB Software Library (FSL v6.0.3)^1^. Firstly, DWI images were corrected for motion artifacts and eddy current distortions. The affine transformations were used to register each volume and were applied to rotate gradient vectors. Then, the first *b*_0_ volume of each subject was extracted and skull stripped, creating a brain mask applied to the remaining volumes in order to remove non-brain structures.

Tensor fitting and the scalar map computation steps were performed with DTIFIT, included in the FDT toolbox. In this step, a diffusion tensor model is fitted at each voxel and scalar maps of FA and MD, as well as eigenvector and eigenvalue maps, were generated. AD was defined as the principal diffusion eigenvalue, and RD was computed using the mean of the second and third eigenvalues.

### Tract-Based Spatial Statistics

Voxel-wise analyses of the scalar maps between subjects were performed using tract-based spatial statistics (TBSS) procedures (Smith et al., 2006), also part of FSL. To remove potential outliers from the tensor fitting, all FA templates were slightly eroded and the end slices were zeroed. Afterward, all the FA templates were non-linearly registered into a 1-mm × 1-mm × 1-mm standard space. This step was performed by non-linearly registering each subject’s FA template to each other to find the “most representative one” (i.e., the one that requires the least warping to align all images), subsequently used as the study-specific target image. Next, the selected target image was affine transformed into the Montreal Neurological Institute (MNI) 152 standard space, and each subject’s FA template was transformed into this standard space by combining the non-linear transformation to the study-specific target with the affine transformation into the MNI space. Then, the FA templates of all subjects were averaged and the resulting image skeletonized. After visual inspection of the skeletonized image, we thresholded it at 0.35 to remove from the skeleton regions encompassing other tissues, such as gray matter or cerebrospinal fluid (CSF). Finally, all scalar maps (FA, AD, MD, and RD) were projected into this FA skeleton using the same transformation applied to the FA templates.

### Statistical Analysis

#### Demographic and Clinical Data

Statistical analyses of the demographic and clinical data were performed with JASP (version 0.11.1; JASP Team, University of Amsterdam, Netherlands). Comparisons between the groups of responders and non-responders were performed using non-parametric Mann–Whitney tests (*U*), given the small unpaired number of participants included in each group (Pett, 2016), and chi-squared tests (χ^2^) for categorical variables. *P*-values under 0.05 were considered statistically significant. The effect size was computed using rank-biserial correlation (*r*_B_) and Pearson’s phi coefficient (*ϕ*) for the Mann–Whitney (*U*) and chi-squared (χ^2^) tests, respectively.

#### Diffusion Data

Non-parametric permutation methods, employed with the *randomize* tool from FSL (Winkler et al., 2014), were used to analyze the skeletonized maps of FA, AD, MD, and RD.

To investigate differences in the WM microstructure at baseline between future responders and non-responders to paroxetine treatment, we performed a two-sample *t*-test, adjusted for age, sex, and time between assessments (pre- and post-treatment). Five thousand permutations were used for each contrast. Widespread significant differences were detected with threshold-free cluster enhancement (TFCE), and multiple comparisons were corrected using family wise error rate (FWE-R) at α = 0.05 and cluster extent threshold of *K* > 50. Clusters showing significant results were identified using the Johns Hopkins University WM Tractography atlas and dilated with the *tbss_fill* tool (included in FSL) for visualization purposes. Additional analyses were performed using IBM^®^ SPSS^®^ Statistics (version 27; IBM Corp., Armonk, NY, United States) to investigate whether the mean global values of the skeletonized maps of FA, AD, MD, and RD predict paroxetine response (see Supplementary Material).

## Results

### Demographic and Clinical Characterization of Groups

Of the 20 participants who completed the posttreatment assessment (6–12 weeks after initiating treatment), 60% (*n* = 12) were classified as responders and 40% (*n* = 8) as non-responders based on the predefined criteria (≥50% reduction in the HDRS score). Table 2 shows the demographic and clinical characterization for both groups pre- and post-treatment.

**TABLE 2:** Description of the demographic and clinical data from responders (*n* = 12) and non-responders (*n* = 8) before and after 6–12 weeks of treatment.

	**Responders (*n* = 12)**		**Non-responders (*n* = 8)**	
	**Pre**	**Post**	**Pre**	**Post**
Age (years)	36.50 (14.50)	–	45.50 (27.25)	–
Sex (male/female)	4/8	–	2/6	–
Education (years)	12.00 (8.00)	–	9.00 (7.75)	–
Time between assessments (weeks)	9.00 (1.50)	–	7.00 (1.25)	–
HDRS score	18.00 (19.50)	3.50 (7.75)	24.50 (6.25)	16.00 (8.75)
HARS score	21.00 (21.75)	4.50 (18.50)	24.50 (10.50)	20.50 (12.50)
PSS-10 score	28.50 (6.50)	17.50 (10.75)	29.50 (8.75)	24.50 (6.50)
BSSI score^a^	0.00 (3.50)	0.00 (3.25)	6.00 (8.50)	4.00 (12.25)

*Data are presented as median (interquartile range).*HDRS*, Hamilton Depression Rating Scale; *HARS*, Hamilton Anxiety Rating Scale; *PSS-10*, 10-item Perceived Stress Scale; *BSSI*, Beck Suicidal Ideation Scale. ^*a*^One responder with missing data pretreatment.*

No significant differences between groups were found regarding age (*U* = 55.50, *p* = 0.589, *r*_B_ = 0.156), sex [χ^2^(1) = 0.159, *p* = 0.690, *ϕ* = 0.089], and education (*U* = 39.00, *p* = 0.509, *r*_B_ = −0.188) and the HDRS (*U* = 48.50, *p* = 1.00, *r*_B_ = 0.010), HARS (*U* = 53.00, *p* = 0.728, *r*_B_ = 0.104), PSS-10 (*U* = 49.50, *p* = 0.938, *r*_B_ = 0.031), and BSSI (*U* = 67.00, *p* = 0.053, *r*_B_ = 0.523) scores at baseline. However, time between the pre- and post-treatment assessments was significantly different between groups (*U* = 22.00, *p* = 0.044, *r*_B_ = −0.542), showing that non-responders (median = 7.00, interquartile range = 1.25) were evaluated earlier than the responders (median = 9.00, interquartile range = 1.50).

After 6–12 weeks of treatment with paroxetine, the responders showed a significant decrease in the HDRS (*U* = 84.00, *p* = 0.006, *r*_B_ = 0.750), HARS (*U* = 77.00, *p* = 0.027, *r*_B_ = 0.604), and PSS-10 (*U* = 75.00, *p* = 0.041, *r*_B_ = 0.563) scores. No significant differences were found in the BSSI scores between groups posttreatment (*U* = 70.00, *p* = 0.076, *r*_B_ = 0.458).

### Pretreatment Differences in the WM Microstructure Associated With Response

**FIGURE 2:**
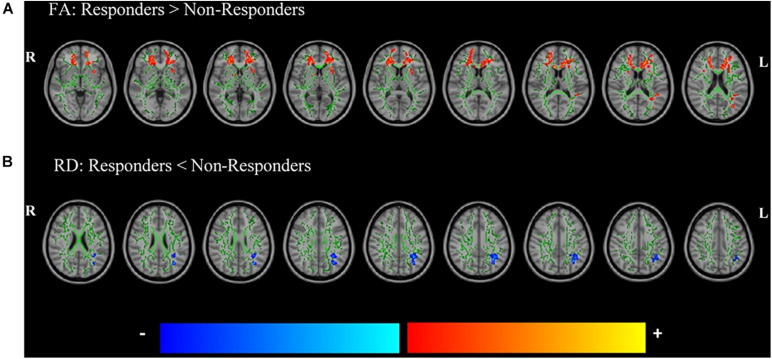
Significant differences in the fractional anisotropy (FA) **(A)** and radial diffusivity (RD) **(B)** maps between future responders and non-responders to paroxetine treatment controlled for time between assessments, age, and sex. Responders had increased FA and decreased RD compared to non-responders. *Red*–*yellow* voxels indicate a significant increase in FA, whereas *blue*–*light blue* voxels indicate a significant decrease in RD in future responders compared with non-responders to paroxetine treatment. Significance threshold was set to *p* < 0.05 [family wise error (FWE) corrected for multiple comparisons]. The white matter (WM) skeleton (represented in *green*) is superimposed on a T1-weighted Montreal Neurological Institute (MNI) template.

Statistically significant differences between groups were found in the FA and RD maps (Table 3 and Figure 2). Responders showed a significant increase in the FA maps compared with non-responders to paroxetine treatment in clusters including forceps minor, bilateral SLF, and the left fronto-occipital fasciculus. A significant decrease in the RD maps was found in responders when compared to non-responders in the left SLF. Results of the investigation of the predictive value of skeletonized maps suggest that FA is the better measure to discriminate responders from non-responders (see Supplementary Material).

**TABLE 3:** White matter tracts with significant differences in FA and RD between future responders and non-responders to paroxetine treatment after controlling for age, sex, and time between assessments.

**WM tract**	**MNI coordinates at signal peak**			**Cluster size**	***p*-values (FWE corrected)**
	***x***	***y***	***z***		
**FA: responders > non-responders**					
Forceps minor	−15	1	30	2,601	0.015
Forceps minor	20	40	11	1,445	0.024
L superior longitudinal fasciculus	−34	−65	26	2,306	0.018
L superior longitudinal fasciculus	−29	7	40	166	0.042
L inferior fronto-occipital fasciculus	−29	12	−1	190	0.042
R superior longitudinal fasciculus	35	16	28	124	0.048
**RD: responders < non-responders**					
L superior longitudinal fasciculus	−31	−60	33	269	0.041

*The WM tracts were identified using the Johns Hopkins University WM Tractography atlas.*L*, left; *R*, right; *WM*, white matter; *MNI*, Montreal Neurological Institute; *FWE*, family wise error, *FA*, Fractional anisotropy; *RD*, radial diffusivity. *p* < 0.05 (FWE corrected), *K* > 50.*

## Discussion

Our study investigated whether paroxetine treatment response was associated with alterations in the WM integrity at baseline in a sample of drug-naive MDD patients. Response was predefined as a 50% reduction in HDRS after 6–12 weeks of treatment. We showed (Figure 2 and Table 3) that, at baseline, responders had higher FA in the forceps minor and SLF and a decreased RD in SLF than did non-responders to paroxetine treatment after controlling for age, sex, and time between assessments. No significant differences were found between groups regarding any demographic and clinical variables.

More than one-third of patients (40%) did not respond to the antidepressant treatment. The pattern of alterations in the WM microstructure found for this group of patients (decreased FA and increased RD) is consistent with previous studies (Vasavada et al., 2016; Tatham et al., 2017; Davis et al., 2019), pointing to a disruption in WM integrity in non-responders. Differently, other studies have reported higher FA in fronto-limbic WM tracts associated with non-response and remission to antidepressant treatment (Taylor et al., 2008; Korgaonkar et al., 2014). However, depression is a very heterogeneous disorder (Monroe and Anderson, 2015), and these apparent contradictory results could be explained by the nature of depression or the use of different samples of MDD patients (with different ages, treatment choices, or antidepressant washout periods), or even different methodological choices regarding data analysis.

The forceps minor and SLF were the two major tracts associated with paroxetine treatment response in our study. The forceps minor is a commissural fiber tract that connects the frontal lobes of both hemispheres through the genu of the corpus callosum (Peltier et al., 2010; Voineskos et al., 2010). Decreased FA in this WM tract was previously associated with non-response to ketamine (Vasavada et al., 2016), suggesting (together with our results) that forceps minor disruption might be a potential biomarker for non-response to antidepressant therapy. Interestingly, deep brain stimulation (DBS) of subcallosal cingulate cortex WM, including specific WM tracts, such as the forceps minor, led to a quicker and stable clinical response in treatment-resistant depression (Riva-Posse et al., 2014; Howell et al., 2019). Overall, these findings might indicate that forceps minor disruptions are not only a potential biomarker for antidepressant non-response but also a potential therapeutic target for stimulation therapies.

The SLF is an association fiber tract connecting the frontal, temporal, and parietal lobes (Schmahmann and Pandya, 2009). It has been described as a key component in the pathophysiology of depression (Murphy and Frodl, 2011), and alterations in its WM microstructure have been linked to depression severity (Lai and Wu, 2014). Both reduced FA in the SLF and forceps minor have been associated with treatment-resistant depression (de Diego-Adeliño et al., 2014). Moreover, FA in the SLF together with the other WM tracts predicted non-remission to SSRIs in 15% of patients, with 84% accuracy, suggesting that more than one tract might be required to predict treatment response effectively (Korgaonkar et al., 2015).

Despite the promising findings of this study, there are several limitations that need to be considered in their interpretation. Firstly, replication in a larger sample is warranted to validate our results. Secondly, our findings only point to a possible association between response to paroxetine treatment and alterations in WM microstructures, given the absence of a control group including untreated depressed patients in this study. Future studies should include a control group with no treatment to validate our results and to allow attributing the alterations in the WM microstructure to paroxetine treatment response. Thirdly, the time between assessments was different between the responders and non-responders, but this variable was included in the DTI analyses as a covariate. Moreover, the inclusion of patients only following paroxetine treatment hampers the generalization of our findings to non-response to other antidepressants. It would be interesting to compare different antidepressants in order to establish whether there are common and specific alterations in the WM microstructure associated with response, which could allow better and personalized treatment. Another limitation was the use of a 1.5-T MRI scanner for the DTI acquisitions, which has a lower signal-to-noise ratio compared to 3-T MRI scanners (Lee and Shannon, 2007). Furthermore, tractography analyses of the forceps minor and the SLF could be performed in future studies to characterize them with higher anatomical resolution.

In conclusion, our study showed that responders to antidepressant treatment with paroxetine present statistically significant differences in the WM microstructure in the forceps minor and the SLF tracts when compared to non-responders. These findings, together with previous literature, pave the way for new studies addressing the potential use of these DTI measures as pretreatment markers of antidepressant response.

## Data Availability Statement

The raw data supporting the conclusions of this article will be made available by the authors, without undue reservation.

## Ethics Statement

The studies involving human participants were reviewed and approved by the Ethics Committees of University of Minho and Hospital de Braga. The patients/participants provided their written informed consent to participate in this study.

## Author Contributions

JB and NS conceived the study. JB recruited the participants. RV and CP-N organized the database and schedule and performed the assessments. JR, AC, RM, SF, and PM performed the MRI acquisitions. RV and AC performed the MRI data pre-processing and data analysis. RV wrote the first draft of the manuscript. All authors contributed to the following and final versions of the manuscript.

## Conflict of Interest

The authors declare that the research was conducted in the absence of any commercial or financial relationships that could be construed as a potential conflict of interest.

## Footnotes

1 https://fsl.fmrib.ox.ac.uk/fsl/
