# Supplementary material for: White Matter Microstructure Alterations Associated With Paroxetine Treatment Response in Major Depression
Source: Front Behav Neurosci. 2021 Jul 22;15:693109. doi: 10.3389/fnbeh.2021.693109 (PMC8341904; doi:10.3389/fnbeh.2021.693109)
Supplement: Supplementary file 1 [file Data_Sheet_1.pdf]

Supplementary Material

1 Supplementary methods

1.1 Statistical analysis

Additional analyses were performed to investigate whether the mean global values of the skeletonized maps of FA, AD, MD and RD predict paroxetine antidepressant response. Global mean values of FA, AD, MD and RD were extracted from each one of the DTI skeletonized maps. Multiple linear regressions were performed between each global mean value of FA, AD, MD and RD and age, sex and time between assessments in order to estimate the variance explained by the 3 predictors. From each model, the standardized residuals were extracted to use in further analyses. Then, receiver operator characteristics (ROC) analysis as the area under the curve (AUC) was performed for each one of the skeletonized maps (using the standardized residuals of FA, AD, MD and RD) in order to assess their accuracy to discriminate responders and non-responders. AUC results were interpreted according to Hosmer and Lemeshow (2000): ≤ .50 no discrimination;].50-.70[weak discrimination; [.70-.80[acceptable discrimination; [.80-.90[good discrimination; and ≥ .90 exceptional discrimination. Binomial logistic regressions were performed using the measures showing good discrimination as predictors and group (responders and non-responders) as outcome.

2 Supplementary results

2.1 Predictors of antidepressant response

ROC analyses were performed for each one of skeletonized maps of FA, AD, MD and RD. The corresponding curves of FA (AUC=.86, 95% confidence interval (CI) .70-1.0), AD (AUC=.54, 95% CI .26-.83), MD (AUC=.36, 95% CI .11-.62), RD (AUC=.20, 95% CI .26-.83) are displayed in Supplementary Figure 1. Given that FA was the only measure that showed good discrimination accuracy according to Hosmer and Lemeshow (2000), a binomial logistic regression analysis was employed using only the standardized residuals of FA as predictor. The binomial logistic regression model was statistically significant ($\chi^2(1)=9.25$, $p=.002$), fitted well the data (Hosmer–Lemeshow test, $\chi^2(8)=4.52$, $p=.807$), and explained 50% (Nargelkerke R^2) of the variance in the response to paroxetine. This model classified accurately 80% of the cases (specificity: 75%; sensitivity: 83.3%). An increased FA was significantly associated with an increase likelihood to respond to paroxetine antidepressant treatment (Wald's $\chi^2(1)=4.04$, $p=.044$, odds ratio (OR)=13.83, 95% CI 1.06-179.9). Summary results of the logistic regression model are displayed in Supplementary Table 1. These results showed be interpreted very carefully and need further validation given the small sample size.

3 Supplementary Figures and Tables

3.1 Supplementary Figures

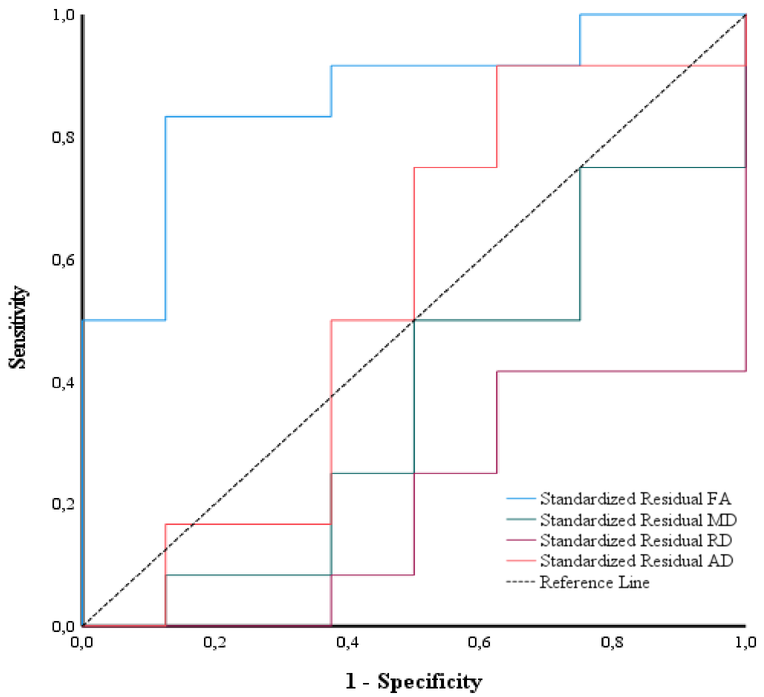

Supplementary Figure 1. Receiver operator characteristics curves of classifications between responders and non-responders for fractional anisotropy, axial, mean and radial diffusivity.

3.2 Supplementary Tables

Supplementary Table 1. Summary results of the logistic regression model.

Predictors	Overall model summary		Model parameters		
	Nagelkerke R^2	*p*	β	SE	*p*
**FA**	.50	<.001	2.63	1.31	.04
**Constant**			.46	.59	.43

Note: FA, standardized residual fractional anisotropy; SE, standard error.

4 References

Hosmer, D.W, and Lemeshow, S.L. (2000). Applied Logistic Regression. 2nd Edition. New York, NY: Wiley.
